# Frailty Is Associated with Impaired Diabetic Foot Ulcer Healing and All-Cause Re-Hospitalization

**DOI:** 10.1007/s12603-022-1726-7

**Published:** 2022-01-22

**Authors:** Giuseppe Maltese, G. Basile, H. Meehan, M. Fuller, M. Cesari, N. Fountoulakis, J. Karalliedde

**Affiliations:** 1 Department of Diabetes and Endocrinology, Epsom and St Helier University Hospitals, Surrey, Epsom, UK; 2 Unit of Geriatric Medicine, Department of Clinical Experimental Medicine, University Hospital of Messina, Messina, Italy; 3 Department of Obstetrics and Gynaecology, Queen Charlotte and Chelsea Hospital, London, UK; 4 Department of Physiotherapy Guy's and St Thomas'NHS Foundation Trust, London, UK; 5 Geriatric Unit, IRCCS Istituti Clinici Scientifici Maugeri, University of Milan, Milan, Italy; 6 Unit for Metabolic Medicine, School of Cardiovascular Medicine & Sciences, Faculty of Life Sciences, King's College London, 150 Stamford Street, SE1 9NH, London, UK

**Keywords:** Diabetic foot ulcers, diabetes, frailty, outcomes

## Abstract

**Background:**

Diabetic Foot Ulcers (DFUs) are a common and feared complication of type 1 and type 2 diabetes. People with DFUs often present a significant clinical complexity due to multimorbidity, frailty, polypharmacy, and disabling conditions. Frailty, defined using the accumulation of health deficits model, has shown to predict worsening health status, hospitalizations, and death in older persons. There are no clinical studies, to date, that have examined the prevalence and effect of frailty on DFUs outcomes. The aim of our study was to explore the impact of frailty on DFUs healing and re-hospitalization in a cohort of patients hospitalized with DFUs.

**Design:**

prospective cohort study.

**Setting and Participants:**

The frailty status of 76 consecutive hospitalized patients with DFUs was assessed by using the Frailty Index (FI).

**Measurements:**

The primary outcome was the non-healing of the DFU. Secondary outcome was re-hospitalization events (for any cause) within 6 months from hospital discharge. Frailty was defined as FI>0.25.

**Results:**

Out of 76 patients (median age 65 years, range 31–84), 56 (74%) were frail. At six months, 81.5% of frail patients had nonhealing of the DFU compared to 55% in non-frail patients (p=0.02). The rate of of re-hospitalization was also higher in frail compared to non-frail (90.3% vs 54%, respectively; p=0.01) patients. In multivariable analyses, frailty was significantly associated with a more than fivefold increased risk of DFU non-healing [odds ratio 5.54 (95% confidence interval 1.28–23.91), p=0.02]. Similarly, re-hospitalization was also significantly higher in frail patients compared to the non-frail ones.

**Conclusions:**

In hospitalized patients with DFUs, frailty was highly prevalent. Frailty emerged as an independent risk factor for DFU non-healing and re-hospitalization events. Patients with DFUs require a comprehensive assessment of their frailty status which would enable personalization of their management and interventions.

## Introduction

Diabetic foot ulcers (DFUs) represent a feared disabling complication of diabetes with a lifetime risk of nearly 25% and a devastating impact on the person's quality of life, morbidity, and mortality (1). Patients with DFUs have a high prevalence of comorbidities and are exposed to accelerated ageing, further enhancing their frailty status (2). Frailty is a condition defined by an excess of vulnerability to endogenous and exogenous stressors, exposing the person to an increased risk of adverse outcomes, including disability, recurrent hospitalizations, and death (3, 4). It has been reported that the prevalence of frailty might range between 27 and 80% in people aged 70 years and older. However, these figures strongly dependent on the care setting and have rarely been explored in specific inpatient settings (5, 6). Among the different operational models of frailty available in the literature, one of the best established and validated is the Frailty Index (FI) proposed by Rockwood and Mitnitski, relying on the assumption that health deficits tend to accumulate with aging (7). The FI directly derives from the results of the clinical assessment of the individual, summarizes in an objective and reproducible way the complexity of the individual, providing an objective surrogate of his/her biological age (8). As such, the FI has unsurprisingly shown to be associated with the risk of adverse outcomes in older persons across clinical settings (Rockwood and Mitnitski, 2007, Theou, 2013, Dent, 2014).

To date, there are no clinical studies that have examined the prevalence and the effect of frailty on DFUs outcomes. Here, we explore in a cohort of inpatients with DFUs, the clinical impact of frailty, measured using the FI, on wound healing and rehospitalization over a 6-month post-discharge period.

## Methods

Data are from a prospective cohort study enrolling 76 consecutive inpatients with DFUs, admitted to St. Thomas' Hospital between October 2015 and August 2016. This study was conducted as part of a service improvement project. Informed consent was obtained from each participant. The only exclusion criterion applied in the study was the presence of severe cognitive impairment (measured by using the Short Portable Mental Status Questionnaire). Frailty was assessed 24 hours after the hospital admission, using a FI designed according to the above-mentioned model of health deficits accumulation and adopting the standardization procedure described by Searle and colleagues (11). The FI was based on 42 dichotomous variables capturing a broad spectrum of health deficits and including chronic diseases, symptoms, disabilities in daily activities, psychological issues, and laboratory abnormalities. Each deficit was scored 1 when present or 0 when absent. The FI was calculated as the number of deficits presented by the individual, divided by the total number of deficits considered in the evaluation (i.e., n=42). Thus, the FI values could range from 0 (no deficit is present) to 1 (all deficits are present), with higher scores indicating a more severe degree of frailty. In agreement with standard practice (Joseph, 2014, Rockwood et al., 2007, Wou, 2013), a cut-point of FI > 0.25 was chosen to define the presence of frailty.

The Patient Health Questionnaire (PHQ-9) was used to assess patients' mood. Minor amputations were defined as amputations below the ankle and major amputations as through and above the ankle. Data on clinical history, past medical history, clinical and biochemical measures were obtained from electronic medical records. Peripheral neuropathy was defined as an altered 10-g Semmes-Weinstein monofilament test. Hypertension was diagnosed if one of the following conditions was present: systolic blood pressure ≥140/90 mm Hg or patient taking at least one anti-hypertensive agent. Peripheral artery disease was defined as the absence of ≥2 peripheral pulses or ≥1 significant stenosis (≥50%) on a duplex scan.

Following the discharge, patients were seen in the foot clinic on a weekly basis until the wound was healed. Patients received treatment, including offloading as per standard care. Wounds with callus and necrotic material were debrided and specimens collected for culture. Antibiotic therapy was prescribed when appropriate.

The primary outcome was non-healing of DFU at six months, defined as no evidence of healing on clinical examination, and/or re-occurrence at six months. The secondary outcome was re-hospitalisation events for any cause within six months from time of hospital discharge.

### Statistical analyses

Descriptive statistics were used for the analysis of demographic and clinical features of the cohort. Between-group differences were compared by unpaired t test (for continuous parametrically distributed variables) and Mann-Whitney test (for continuous non-parametrically distributed variables). A χ2 test was used to compare categorical variables between groups. Data are given as mean ± SD, the percentage for categorical variables, or median and IQR for variables not normally distributed. A two-tailed p-value <0.05 was considered significant. To explore whether frailty was independently associated with the study outcomes, multiple logistic regression analyses were performed. Odds ratios (ORs) and 95% confidence intervals (CI) are reported. A hierarchical block entry method of entering predictor variables to build the logistic regression model was used. Statistical analyses were performed with SPSS version 25.0 (SPSS, Chicago, IL, USA).

## Results

A total of 76 consecutive patients (Type 1 Diabetes n=8, Type 2 Diabetes n=68) were prospectively enrolled in this study. The majority of patients were men (80%), and the median age was 65 (range 31–84) years. The mean FI was 0.32 (standard deviation, SD 0.13), and 54 patients (71%) could be considered frail as presenting a FI higher than 0.25. The median length of hospitalization for the DFU was 16 (range 3–123) days.

Participants were divided into two groups, non-frail and frail (Table 1). Frail patients had lower estimated glomerular filtration rate (eGFR), and higher prevalence of PAD, use of medications, and had a longer DFU duration. There were no other significant differences between the two groups.

**Table 1:** Baseline demographic, clinical, and laboratory characteristics of 76 patients with diabetic foot ulcers, non-frail and frail

**Variable**	**Non-frail (N=22)**	**Frail (N=54)**	**p-value**
Age (years)	61.32±8.0	65.3±10.5	0.11
Gender (male %)	19 (86.4)	42 (77.8)	0.53
Peripheral vascular disease n (%)	8 (38.1)	41 (77.4)	0.02
Peripheral neuropathy n (%)	13 (59.1)	41 (77.4)	0.16
eGFR (ml/min)	81.2±34.7	63.5±29.3	0.03
HbA1c (%) (mmol/mol)	9.6±2.8	9.3±2.7	0.64
Ischemic heart disease n (%)	3 (13.6)	19 (35.8)	0.09
Heat failure n (%)	2 (9.1)	11 (20.8)	0.32
Arrhythmia n (%)	2 (9.1)	14 (26.4)	0.13
Obesity (BMI ≥30) n (%)	11 (50)	23 (43.4)	0.62
Cerebrovascular disease n (%)	0 (0)	7 (13.2)	0.98
Cognitive impairment n (%)	1 (4.5)	9 (17%)	0.26
Depression n (%)	3 (13.6)	18 (34)	0.94
Diabetic retinopathy n (%)	8 (36.4)	31 (58.5)	0.13
Polypharmacotherapy n (%)	11 (50)	47 (88.7)	0.001
Smoking n (%)	6 (27.3)	18 (34)	0.79
Ulcer duration>12 weeks n (%)	6 (27.3)	35 (66)	0.004


Abbreviations: eGFR= estimated glomerular filtration rate

DFUs were predominantly neuro-ischemic and located in the dorsal, lateral, and medial feet and toes. During the 6-month follow-up, six patients died in the frail group and no one in the non-frail group.

The total number of patients experiencing an amputation was 39. Of these, 32 and 7 had a minor and a major amputation, respectively. Frail patients had a higher risk of poor DFU healing than those with a FI ≤0.25 (81.5% vs. 55%; P=0.02). The rate of re-hospitalization was also higher in frail patients compared to the non-frail ones (90.3% vs. 54%, p=0.01).

As detailed in Table 2, frailty was the only variable significantly different between the two groups for the two outcomes of interest. In a multivariable logistic regression analysis, with the FI considered a dichotomous variable, patients with frailty were at increased risk of developing poor DFU healing (OR 5.54, 95%CI 1.28–23.91, p=0.02) and re-hospitalization (OR 17.52, 95%CI 2.65–116) p=0.003), after adjustment for other established and potential risk factors (Table 3).

**Table 2:** Comparisons between patients with DFU healing and DFU non-healing and those with and without rehospitalisation

	**Healing**	**Non-healing**	**p-value**	**Rehospitalisation**	**No rehospitalisation**	**p-value**
Age (years)	62.7±10.5	64.1±10.2	0.6	62.7±11.3	64.9±9.5	0.4
Gender (m/%)	85	84.2	0.9	83.8	83.9	0.9
Haemoglobin	110.8±17.2	107.5±15.7	0.4	110±15.5	107.7±18.2	0.6
eGFR (ml/min)	71.3±27.4	65.8±30.4	0.45	67.5±29.6	69.2±33.6	0.8
HbA1c (%)	9.4±3.3	9.7±2.3	0.7	9.2±2.6	9.8±3	0.4
Type of diabetes (T2DM)	92.5	83.7	0.3	86.6	89.2	0.8
Ulcer duration>12 weeks (%)	59	55	0.8	61	51	0.4
Frailty index> 0.25 (%)	55	81.5	0.023	90.3	54	0.001


Abbreviations: eGFR= estimated glomerular filtration rate, T2DM= type 2 diabetes mellitus

**Table 3:** Multivariable logistic regression analysis of variables associated with DFU non-healing and re-hospitalization*

**DFU Non-healing**	**OR**	**95% CI**	**P**
Age	0.99	0.94–1.05	0.84
Gender	0.80	0.19–3.49	0.77
Smoking	1.29	0.38–4.43	0.68
> 5 drugs per day	0.39	0.08–1.94	0.25
FI >0.25	5.54	1.29–23.91	0.02
Re-hospitalization	**OR**	**95% CI**	**P**
Age	0.93	0.86–1.00	0.04
Gender	1.38	0.32–6.00	0.67
Smoking	2.84	0.64–12.5	0.17
> 5 drugs per day	1.50	0.28–8.09	0.64
FI >0.25	0.06	0.01–0.38	0.003


No other variable other than the ones depicted in the table were included in the multivariable analyses; Abbreviations: DFU= Diabetic Foot Ulcer, FI= Frailty Index

## Discussion

Our study in patients with DFUs is the first to report the negative impact of frailty (as evaluated using the FI) on poor DFUs outcomes and rehospitilizations. We show that frailty is very common in this relatively young cohort (median age 65) of patients with a prevalence of 71%. This prevalence is significantly higher than what is reported in the literature, even in much older non-diabetic populations (6, 15). These results confirm that frailty is not exclusively a condition of old age, but can exist among young individuals exposed to specific conditions accelerating the aging process. DFUs and their associated complications (e.g., amputations) can indeed be seen as major contributors to the fragilization of the individual, leading to disabling conditions and adverse outcomes by feeding the vicious cycle of frailty. Interestingly, previous studies have found that frailty is associated with poorer outcomes also at a young age (16).

We also report here that frailty predicts non-healing of DFUs. Sarcopenia, neuropathy, and inflammatory mechanisms may represent some of the underlying shared mechanisms. There is established evidence that chronic conditions, such as heart failure, renal impairment, and depression, interfere with the healing of DFUs (Rhou, 2015, Game et al., 2013, Vedhara, 2010). The cumulative effect of multiple pathologies (potentially accounting for frailty) on DFUs has not been evaluated. The high prevalence of frailty may explain why some DFUs still fail to improve despite removing barriers to healing (e.g., infection, edema, reduced blood supply). Polypharmacy, another critical issue in frail individuals, might also have a direct effect on tissue repair. In fact, drugs like steroids, nicorandil, diuretics, antihypertensive agents, and immunosuppressants are known to slow down wound healing (20). It is also possible that the exhausted homeostatic reserves characterizing the frail individual may play a role in the non-healing of the DFUs.

Our findings on the increased risk of rehospitalisation in patients with DFUs and frailty are consistent with the literature from other studies in older non-diabetic cohorts (7, 21, 22).

We also observed a higher prevalence of frailty in men than women, which is opposite to what is generally reported in the literature (23, 24). A possible explanation may be that DFUs are more common in men (who are also at higher risk of amputations) than women.

We did not observe in multivariable analyses a significant independent association between frailty and mortality in our cohort. This finding contrasts with recent studies of hospitalized older patients, where the FI significantly correlated with the risk of in-hospital death and 1-year mortality (25). The relatively younger age of our cohort as well as the limited sample size may explain why we did not observe a similar increased risk of mortality.

The FI provides an accurate clinical instrument to identify frail patients, and potentially an opportunity to person-tailor post-discharge interventions. The FI is easy to administer at the bedside, does not require any special equipment, and is predictive of adverse clinical outcomes. Importantly, its quantitative and not qualitative nature makes it easily applicable and adaptable to routine clinical practice.

The early identification of patients at risk of adverse events and assessing the severity of frailty have important clinical implications. Several studies have demonstrated that targeted post-discharge care planning using the FI can improve the patient's outcomes (Naylor, 1994, Rich, 1995, Naylor, 1999, Jack, 2009).

We acknowledge several limitations of our study. First, the number of patients recruited was relatively small, and the DFUs size was not directly measured. We cannot exclude that the size of the lesion may have impacted on our findings. However, as the DFU might be considered a health deficit per se, it is likely that the FI is highly correlated with the size and severity of the lesion. Second, we did not evaluate the frailty status at discharge. Numerous studies have shown that hospital admission per se plays a role in subsequent functional deterioration. Finally, we included patients admitted to a tertiary hospital, a regional centre for DFUs treatment. Thus, the results may not be generalizable.

Despite these limitations, our data highlight the impact of frailty on patients with DFUs. The strengths of our study are the robust evaluation of frailty by using a validated model, the standardized clinical care process, the optimal DFUs evaluation and treatments that patients received, and the long duration of follow-up to assess the relevant outcomes (for both the patient and the healthcare system).

Our findings should foster research into the possible extension of the multidimensional approach to young people with diabetes and DFUs. Indeed, the distinction according to chronological age in these frail patients might be arguable (and even lead to ageistic approaches). In this context, the FI might allow more sound clinical decisions relying on a surrogate of biological age. A better understanding of frailty will enable to improve the individualization of care planning for patients with DFUs.
